# Inflammation increases NOTCH1 activity via MMP9 and is counteracted by Eicosapentaenoic Acid-free fatty acid in colon cancer cells

**DOI:** 10.1038/srep20670

**Published:** 2016-02-11

**Authors:** Chiara Fazio, Giulia Piazzi, Paola Vitaglione, Vincenzo Fogliano, Alessandra Munarini, Anna Prossomariti, Maddalena Milazzo, Leonarda D’Angelo, Manuela Napolitano, Pasquale Chieco, Andrea Belluzzi, Franco Bazzoli, Luigi Ricciardiello

**Affiliations:** 1 Department of Surgical and Medical Sciences, University of Bologna, Bologna, Italy; 2 Center for Applied Biomedical Research (CRBA), S.Orsola-Malpighi Hospital, University of Bologna, Bologna, Italy; 3 Department of Food Science, University of Naples “Federico II”, Portici (NA), Italy; 4 Food Quality &amp; Design group, Wageningen University, Wageningen, The Netherlands; 5 Endocrinology Unit S.Orsola-Malpighi Hospital, Bologna, Italy; 6 Gastroenterology Unit S.Orsola-Malpighi Hospital, Bologna, Italy

## Abstract

Aberrant NOTCH1 signalling is critically involved in multiple models of colorectal cancer (CRC) and a prominent role of NOTCH1 activity during inflammation has emerged. Epithelial to Mesenchymal Transition (EMT), a crucial event promoting malignant transformation, is regulated by inflammation and Metalloproteinase-9 (MMP9) plays an important role in this process. Eicosapentaenoic Acid (EPA), an omega-3 polyunsaturated fatty acid, was shown to prevent colonic tumors in different settings. We recently found that an extra-pure formulation of EPA as Free Fatty Acid (EPA-FFA) protects from colon cancer development in a mouse model of Colitis-Associated Cancer (CAC) through modulation of NOTCH1 signalling. In this study, we exposed colon cancer cells to an inflammatory stimulus represented by a cytokine-enriched Conditioned Medium (CM), obtained from THP1-differentiated macrophages. We found, for the first time, that CM strongly up-regulated NOTCH1 signalling and EMT markers, leading to increased invasiveness. Importantly, NOTCH1 signalling was dependent on MMP9 activity, upon CM exposure. We show that a non-cytotoxic pre-treatment with EPA-FFA antagonizes the effect of inflammation on NOTCH1 signalling, with reduction of MMP9 activity and invasiveness. In conclusion, our data suggest that, in CRC cells, inflammation induces NOTCH1 activity through MMP9 up-regulation and that this mechanism can be counteracted by EPA-FFA.

Inflammation has a central role in colorectal cancer (CRC) development and progression^1^. Besides inflammatory bowel diseases (IBD)^2^, chronic exposure of intestinal epithelial cells to pro-inflammatory *stimuli* can be due to various reasons, including infections^3^, microbiota alterations^4^, metabolic disorders and obesity^5^. In CRC, epithelial cells are surrounded by an inflammatory microenvironment highly populated by immune cells, including macrophages, which produce a heterogeneous mix of cytokines, chemokines and growth factors^6^,^7^. There are increasing evidences that specific cytokines, including interleukin-6 (IL-6), chemokine-8 (CXCL8) and Tumor Necrosis Factor-alpha (TNF-α) have a primary role in the pathogenesis of sporadic CRC^8^,^9^,^10^,^11^,^12^. However, in sporadic CRC development and progression the effects of the combined pro-inflammatory mediators, resembling the inflammatory *milieu* within the tumor microenvironment, have not been completely elucidated.

NOTCH receptors are a family of trans-membrane proteins which drive a fundamental and highly conserved pathway involved in the control of cell fate, proliferation, and death^13^. Upon binding with its ligand Delta-like (DLL) or Jagged (JAG), NOTCH receptors undergo subsequent proteolytic cleavages which culminate with the release and nuclear translocation of the active NOTCH Intracellular Domain (NICD), with transcription of NOTCH downstream targets, including a bHLH transcription factor, hairy and enhancer of split-1 *(HES1)*^14^. It is well known that the deregulation of the NOTCH1 signalling cascade plays a pivotal role in several solid tumors, including CRC^15^. Importantly, while DLL1-mediated NOTCH1 signalling is required for intestinal stem-cell homeostasis^16^, the binding to JAG1 is associated with colon carcinogenesis^17^. It has been recently demonstrated that besides *HES1*, which in turn blocks atonal homolog 1 (*ATOH1*) transcription, as downstream target, NOTCH-regulated ankyrin repeat protein (*NRARP*) transcription level accurately reveals the intestinal activation of NOTCH1^18^. Depending on the CRC settings, a dichotomy for the role of NOTCH1 pathway activation is reported: while in sporadic or hereditary models of CRC NOTCH1 acts as an oncogene and is required for adenoma formation^19^ and cancer progression^20^, in colitis-associated cancer (CAC) NOTCH1 seems to have a tumor suppressor role and its inhibition is associated with decreased apoptosis^21^. Recent experimental results acknowledge the role of the NOTCH pathway for regulating inflammatory processes^22^. In CRC, the activation of NOTCH1 signalling in response to pro-inflammatory *stimuli* has recently emerged^23^; however, the underlying mechanisms are far from being clarified.

Epithelial to Mesenchymal Transition (EMT) is a process that enables an epithelial cell to undergo multiple biochemical changes, allowing it to assume a mesenchymal phenotype^24^. EMT has been well-documented in multiple cancer models including CRC, where it promotes tumor progression and enhances invasiveness^25^,^26^,^27^,^28^. The initiation and progression of EMT involves distinct signalling pathways and cross-talks^29^. During EMT, changes in gene expression master regulators typically occur, such as in zinc-finger E-box binding 1 (*ZEB1*) and its direct target E-cadherin (*CDH1*) as well as matrix metalloproteinases (MMPs), including *MMP9*^30^,^31^. Among other mechanisms, it is known that the release of inflammatory cytokines by immune cells contributes to EMT^32^,^33^. Recently, a link between MMP9 and NOTCH1 signalling has been demonstrated^21^,^34^. However, the relationship among inflammation, EMT and NOTCH1 remains unclear.

Omega-3 polyunsaturated fatty acids (ω-3 PUFAs), such as Eicosapentaenoic acid (EPA) and Docosahexaenoic Acid (DHA), are natural compounds from oily fish known for their anti-inflammatory properties. The beneficial effects of ω-3 PUFAs against CRC have been widely observed in many *in vivo* studies^35^. Recently, it has been shown that EPA works as a chemopreventive agent in multiple models of colorectal cancer, including familial adenomatous polyposis (FAP) and Colitis-Associated Cancer (CAC), by acting on several molecular pathways^36^,^37^,^38^.

The aim of this study was to determine whether NOTCH1 signalling can be induced by a cytokine-enriched Conditioned Medium (CM) obtained from activated THP-1 macrophages and whether this mechanism could be related to EMT in CRC cells. Moreover, we investigated whether this pathway could be counteracted by the treatment with EPA in a free-fatty acid form (EPA-FFA).

For the first time we show that CM, by increasing MMP9 expression, functions as a strong activator of NOTCH1 signalling. Importantly, we found that EPA-FFA treatment counteracts the inflammation-driven NOTCH1 activation leading to a concomitant decrease of invasiveness. Therefore, our data suggest a possible new protective effect of EPA-FFA in response to inflammation on CRC.

## Results

### PMA-differentiated THP1 produce a Conditioned Medium enriched with pro-inflammatory mediators

In order to obtain a stimulus able to mimic the complexity of the inflammatory microenvironment, we differentiated the THP1-monocytes into macrophages and we collected the supernatant. FACS analysis of PMA-differentiated THP1 cells showed increased expression of CD11b (p = 0.0027) and CD14 (p = 0.0507), which are specific markers of macrophages, compared to untreated cells (Supplementary Fig. S1A).

**Figure 1:**
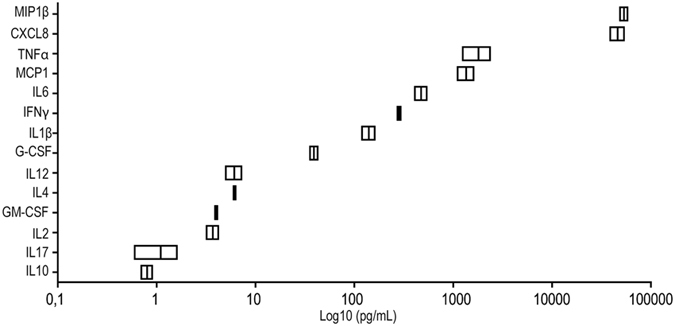
Explorative analysis of inflammatory mediators contained in the Conditioned Medium (CM) from PMA-differentiated THP1 after activation with LPS. Analysis was performed with a multiplex bead-based sandwich immunoassay to detect 14 inflammatory mediators; concentrations (pg/ml) are reported on a log10 scale.

The Conditioned Medium (CM) from LPS-stimulated THP1 included the inflammatory mediators shown in Fig. 1. Moreover, among the 6 most expressed pro-inflammatory mediators, we found that IL-6 (p = 0.0263), CXCL8 (p = 0.004), TNF-α (p = 0.0053) and MIP-1β (p < 0.0001) significantly increased in the supernatant from LPS stimulated-THP1 compared to the unstimulated counterpart, while no differences were found for IFN-γ and MCP-1 (Supplementary Fig. S1B). These data suggest that the PMA-differentiated THP1cells secrete a complex pool of mediators which resembles a multi-inflammatory stimulus.

### The Conditioned Medium promotes NOTCH1 pathway and EMT in CRC cells

**Figure 2:**
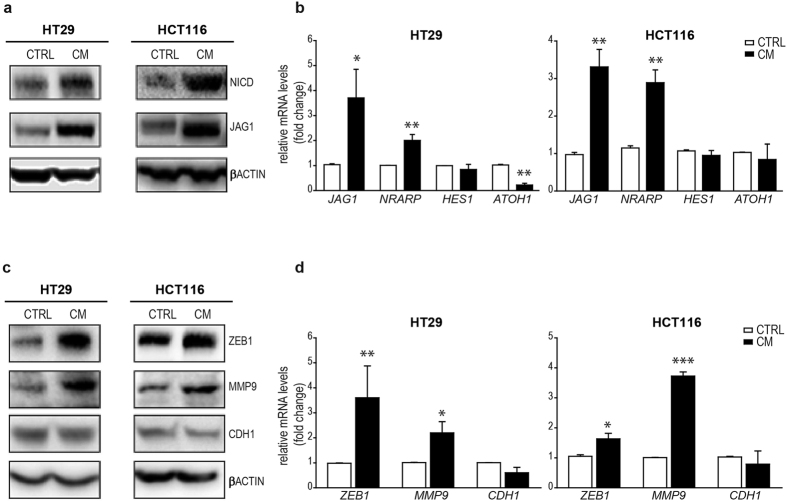
NOTCH1 pathway and EMT markers in HT29 and HCT116 cells under inflammatory conditions. (**a**) Western Blot analyses for NICD and JAG1 in HT29 and HCT116 exposed to CM for 12 h. β-ACTIN was used as housekeeping protein. Each experiment was repeated at least three times. (**b**) qRT-PCR for *JAG1* and NRARP, *HES1* and *ATOH1* in HT29 and HCT116 cells incubated in CM; Statistical significance was calculated on logarithmic transformed values using unpaired t-test, (n = 3, N = 4). *p < 0.05; **p < 0.01; ***p < 0.001. *GAPDH* was used as housekeeping gene. (**c**) Western Blot analyses for ZEB1, MMP9, and CDH1 in HT29 and HCT116 exposed to CM for 12 h. β-ACTIN was used as housekeeping protein. Each experiment was repeated at least three times. (**d**) qRT-PCR for *ZEB1, MMP9* and *CDH1* in HT29 and HCT116 cells incubated in CM. *GAPDH* was used as housekeeping gene. Statistical significance was calculated on logarithmic transformed values using unpaired t-test, (n = 3, N = 4 for HT29 and n = 3, N = 3 for HCT116). *p < 0.05; **p < 0.01; ***p < 0.001.

To determine whether the THP-1 derived CM could drive the activation of NOTCH1 pathway, we incubated CRC cell lines with the CM. Upon 12 h of CM exposure, we found increased protein levels of the Notch1 Intracellular Domain (NICD) in HT29 and HCT116 (Fig. 2a) and LS147T (Supplementary Fig. S2) cell lines. The CM-driven up-regulation of NOTCH1 pathway in HT29 and HCT116 cells was also confirmed at mRNA level by a significant increase of the NOTCH1 target *NRARP* (p = 0.0011 for HT29 and p = 0.0023 for HCT116, CTRL vs CM), and a consensual decrease of *ATOH1* (p = 0.0027 for HT29 and p = n.s for HCT116, CTRL vs CM) together with the increased expression of the NOTCH1 ligand *JAG1* (p = 0.0443 for HT29 and p = 0.0012 HCT116, CTRL vs CM). Interestingly, no variations were observed in the level of the *HES1* transcripts after NOTCH1 activation (Fig. 2b).

To strengthen the concept that a multi-cytokine exposure (rather than the effect of a single inflammatory agent) triggered the activation of NOTCH1 signalling in this model, we also tested the effect of single IL-6, CXCL8 or TNF-α exposures on CRC cells, given their abundance in the CM. Accordingly to our hypothesis, the treatment with single cytokines did not increase the protein level of NICD and JAG1 (Supplementary Fig. S3).

As different cytokines are known to promote EMT in cancer cells^32^,^33^,^39^,^40^ we checked its modulation in our model. In particular, we focused on ZEB1 and its direct target CDH1 and MMP9 because a modulation of NICD in MMP9-overexpressing cells has been recently demonstrated^21^. Compared to the controls, we found increased protein levels of ZEB1 and MMP9 both in HT29 and HCT116 and a concomitant modest reduction of E-Cadherin (CDH1) upon CM exposure in HCT116 (Fig. 2c). These results were confirmed by a significant increase of *ZEB1* (p = 0.008 for HT29 and p = 0.0373 for HCT116, CTRL vs CM) and *MMP9* (p = 0.0224 for HT29 and p < 0.0001 for HCT116, CTRL vs CM) transcripts and by a consensual but not significant decrease of *CDH1* in CM-treated cells compared to the controls (Fig. 2d).

Thus, our results suggest that the inflammatory microenvironment can contextually activate NOTCH1 signalling and EMT in CRC cells.

### NOTCH1 up-regulation depends on MMP9 expression upon inflammation

**Figure 3:**
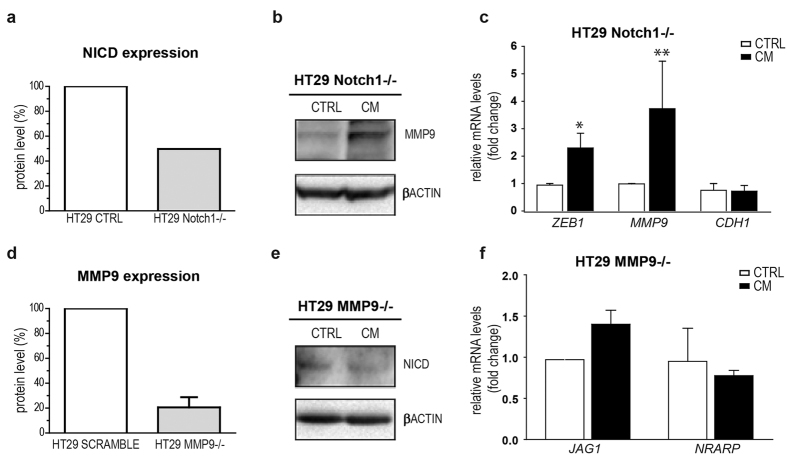
NOTCH1- and MMP9-silenced HT29 cells under inflammatory conditions. (**a**) NICD protein level in HT29 (CTRL) and NOTCH1 stably-silenced HT29 (NOTCH1^−/−^). (**b**) Western Blot analyses for MMP9 in NOTCH1-silenced HT29 cells exposed to CM. Each experiment was repeated three times. (**c**) qRT-PCR for *ZEB1, MMP9* and *CDH1* in NOTCH1 silenced-HT29 cells incubated in CM. Statistical significance was calculated on logarithmic transformed values using unpaired t-test, (n = 3, N = 3). *p < 0.05; **p < 0.01; ***p < 0.001. (**d**) MMP9 protein level in scramble and MMP9 transiently silenced-HT29 (MMP9^−/−^). (**e**) Western Blot analysis for NICD in MMP9-silenced HT29 cells exposed to CM for 12 h. Each experiment was repeated twice. (**f**) qRT-PCR for *JAG1* and *NRARP* in MMP9-silenced HT29 and HCT116 cells incubated in CM, Statistical significance was calculated on logarithmic transformed values using unpaired t-test, (n = 3, N = 2). *p < 0.05; **p < 0.01; ***p < 0.001. GAPDH was used as housekeeping gene.

For a better understanding of the link between pro-inflammatory stimulus, NOTCH1 signalling and EMT modulation, we tested the effect of CM in NOTCH1- or MMP9-silenced HT29 cells (Supplementary Fig. S4). Interestingly, we observed that MMP9 induction upon CM exposure persisted even in absence of NOTCH1 (Fig. 3a,b). Accordingly, when we evaluated the mRNA expression of EMT mediators in CM-treated HT29 NOTCH1^−/−^ cells, we still found a significant up-regulation of *ZEB1* and *MMP9* (p = 0.0371 and p = 0.0336, respectively) in NOTCH1^−/−^ CM vs NOTCH1^−/−^ CTRL (Fig. 3c), suggesting that the induction of the EMT mediators triggered by CM was independent or precedes the NOTCH1 activation.

If MMP9 causes the activation of NOTCH1, it is expected that silencing of MMP9 does not lead to CM-driven NICD overexpression. In order to verify this hypothesis, we transiently silenced MMP9 in HT29 cells (Fig. 3d) and found that the protein level of NICD did not increase upon CM compared to its scramble control (Fig. 3e). Accordingly, in MMP9-silenced cells the transcriptional level of *JAG1* and *NRARP* were not increased by CM stimulation (Fig. 3f). Taken together, our data show that inflammation up-regulates MMP9 which is in turn responsible for the activation of NOTCH1 pathway in CRC cells.

### EPA-FFA treatment counteracts inflammatory-driven NOTCH1 activation

**Figure 4:**
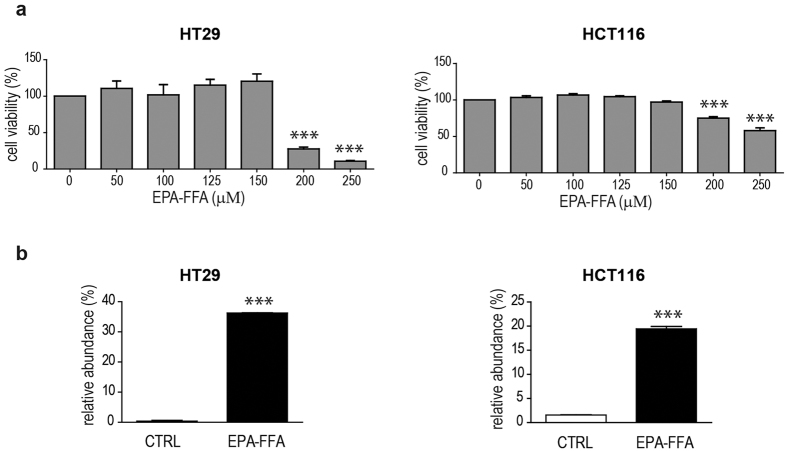
(**a**) Cell viability of HT29 (left panel) and HCT116 (right panel) treated with EPA-FFA. Each experiment was performed in quintuplicate. Concentrations of EPA-FFA lower than 200 μM (HT29) and 150 μM (HCT116) did not cause cytotoxicity (ANOVA p < 0.0001, Dunnet’s test). (**b**) Relative abundance of EPA into the cellular membranes of HT29 (left panel) and HCT116 (right panel) treated or not with 50 uM of EPA-FFA for 72 h ^*^= p < 0.05, ^**^= p < 0.01, ^***^= p < 0.001.

In order to unravel new mechanisms, in this study EPA-FFA was used for investigating its effect on CM-driven NOTCH1 and EMT signalling activation. HT29 and HCT116 were pre-treated with a non-cytotoxic concentration of EPA-FFA (50 μM for 72 h), as previously established by MTT assay (Fig. 4a). We found that the treatment led to a 36% and 19% of EPA incorporation into HT29 and HCT116 cellular membranes, respectively (Fig. 4b). We also optimized a prolonged treatment on HT29 (14 days of EPA-FFA) and observed that the levels of EPA incorporation were similar after 72 h or 14 days of EPA-FFA (Supplementary Fig. S5). Therefore, we decided to incubate our cells with EPA-FFA for 72 h. After EPA-FFA pre-treatment, HT29 and HCT116 were incubated with CM.

**Figure 5:**
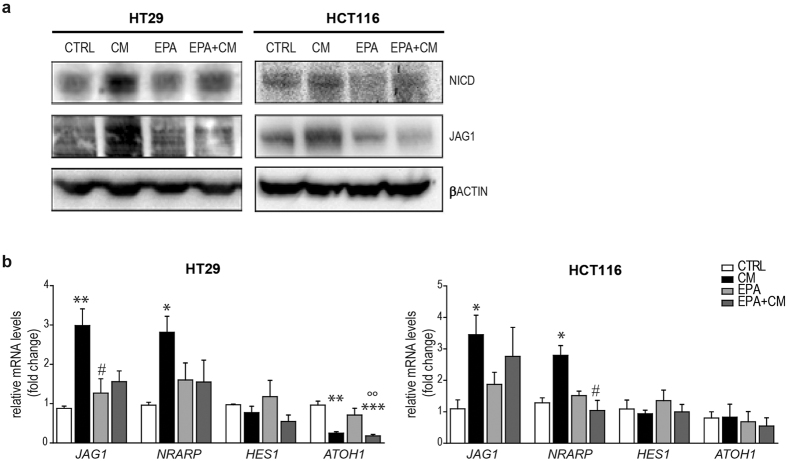
EPA-FFA represses inflammatory-driven NOTCH1 activation. (**a**) Western Blot analyses for NICD and JAG1 in HT29 (left panel) and HCT116 (right panel) in incubated with CM (12 h) and/or pre-treated with EPA-FFA (72 h). β-ACTIN was used as housekeeping protein. (**b**) qRT-PCR in HT29 (left panel) for *JAG1* (ANOVA, p = 0.0085), *NRARP* (ANOVA, p = 0.0440), *HES1* (ANOVA, p = n.s.) and *ATOH1* (ANOVA, p = 0.0006) treated with EPA-FFA (72 h) and/or CM (12 h). qRT-PCR in HCT116 (right panel) for *JAG1* (p = 0.0509), *NRARP* (p = 0.0136), *HES1* (p = n.s.) and *ATOH1* (p = n.s.) in treated with EPA-FFA (72 h) and/or CM (12 h). Data were corrected by logarithmic transformation and analyzed by One-way ANOVA and Tukey’s Test for multiple pairwise comparisons (n = 3, N = 3). *p < 0.05, **p < 0.01, ***p < 0.001 compared to CTRL; ^#^p < 0.05, ^##^p < 0.01, ^###^p < 0.001 compared to CM; °p < 0.05, °°p < 0.01, °°°p < 0.001 compared to EPA-FFA.

Our data show that EPA-FFA was able to reduce the CM-induction of JAG1 and NICD in HT29 and HCT116 cells (Fig. 5a). Accordingly, the EPA-FFA pre-treatment alone did not significantly change the mRNA levels of *JAG1*, *NRARP*, *HES1* and *ATOH1* (p = n.s. CTRL vs EPA) but was able to significantly counteract their increase in both HT29 and HCT116 CM-treated cells, (p < 0.01 and p < 0.05 CTRL vs CM; p = n.s. CTRL vs EPA + CM, HT29 and HCT116 respectively), (Fig. 5b). However, pre-treatment with EPA-FFA did not reverse the profound *ATOH1* down-regulation in HT29 cells (p < 0.001 CTRL vs EPA + CM) obtained after CM incubation.

These findings suggest that EPA-FFA could counteract the activation of NOTCH1 signalling under inflammatory conditions.

### Effect of EPA-FFA on CM-driven MMP9 activation and cell invasion

**Figure 6:**
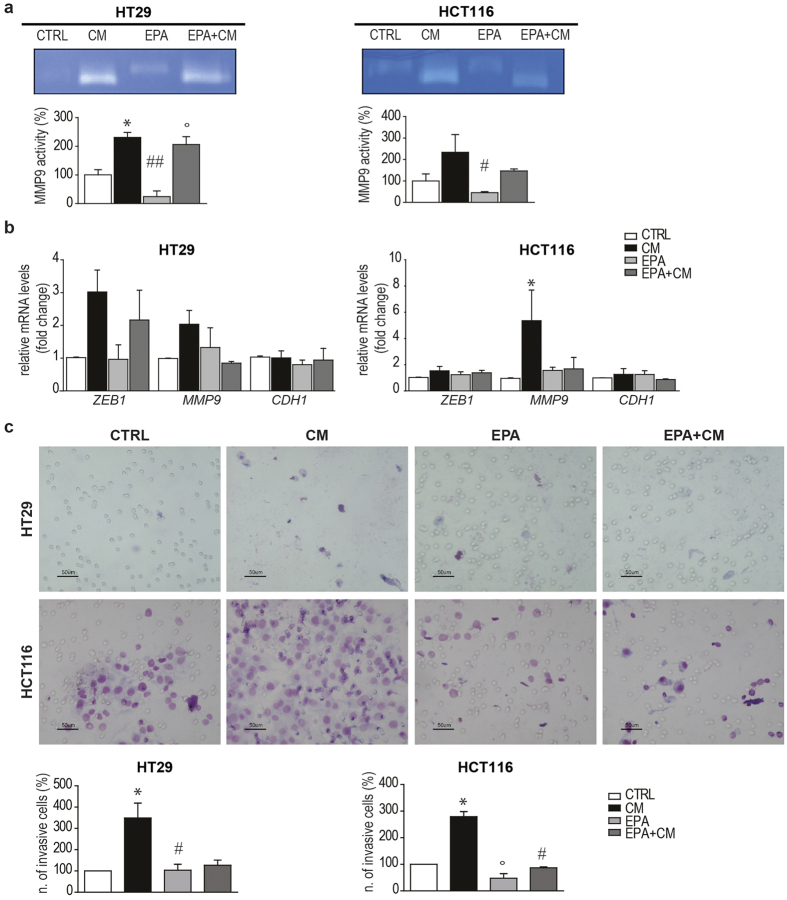
Effect of EPA-FFA on CM-induced MMP9 activation and invasion. (**a**) Gelatin zymography in response to EPA-FFA (72 h) and/or CM (12 h) treatment in HT29 (left panel) and HCT116 (right panel). Bars indicate Mean ± SEM of densitometric values of MMP9 active form normalized to the corresponding CTRL values (ANOVA p = 0.0067 for HT29 and p = 0.0473 for HCT116, (N = 2). (**b**) qRT-PCR for *ZEB1* (ANOVA p = n.s for HT29 and HCT116), *MMP9* (ANOVA p = n.s for HT29 and p = 0.0525 for HCT116) and *CDH1* (ANOVA, p = n.s for HT29 and HCT116) in HT29 and HCT116 EPA-FFA and/or CM treated cells (n = 2; N = 3). (**c**) Matrigel invasion assay of HT29 (upper panel) and HCT116 (lower panel) treated with EPA-FFA (72 h) and/or CM (12 h). Bars indicate Mean ± SEM of number of invasive cells/chamber normalized to the corresponding CTRL values (ANOVA p = 0.0284 for HT29 and p = 0.0007 for HCT116), (n = 5, N = 2). Analyses were performed on logarithmic transformed data. After the ANOVA global test, Tukey’s post hoc test was used for multiple pairwise comparisons. *p < 0.05, **p < 0.01, ***p < 0.001 compared to CTRL; ^#^p < 0.05, ^##^p < 0.01, ^###^p < 0.001 compared to CM; °p < 0.05, °°p < 0.01, °°°p < 0.001 compared to EPA-FFA.

To clarify the role of EPA-FFA on NOTCH1 pathway and in order to evaluate the ability of EPA-FFA to prevent the EMT phenotype led by CM, we assayed MMP9 activity by gelatin zymography on CRC cells treated with CM alone or following a 72 h EPA-FFA pre-treatment. As shown in Fig. 6a, we observed that CM increased MMP9 activity in HT29 and HCT116 (p < 0.05 and p = n.s. CTRL vs CM, respectively). Although not reaching a statistical significance, we also found that pre-treatment with EPA-FFA reduces MMP9 activity in HCT116 (p = n.s. CM vs EPA + CM).

Accordingly, upon EPA-FFA treatment, we found a decreasing trend of mRNA levels for *ZEB1* (p = n.s. in HCT116 and HT29) and *MMP9* (p < 0.05 in HCT116, CTRL vs CM; p = n.s. CTRL vs EPA + CM; p = n.s. in HT29) compared to CM (Fig. 6c). Finally, we evaluated the invasive behaviour of CRC cells treated with CM and/or EPA-FFA. We noticed that CM significantly induced invasiveness in both HT29 and HCT116 (p < 0.05 for both compared to controls) and pre-treatment with EPA-FFA strongly reduced this effect (p = n.s. for both compared to controls), (Fig. 6c).

Taken together our results indicate that EPA-FFA is able to reduce the inflammatory-driven Epithelial to Mesenchymal Transition and prevent invasiveness *in vitro*.

## Discussion

In this study, we demonstrated for the first time that the exposure to a cytokine-enriched Conditioned Medium (CM), obtained from THP1-derived macrophages, up-regulates NOTCH1 signalling in CRC cells, changes Epithelial to Mesenchymal Transition (EMT) markers and induces cell invasion. Notably, we found that NOTCH1 activation depends on MMP9 expression under inflammatory conditions. Importantly, we observed that the treatment with Eicosapentaenoic Acid in the free fatty acid form (EPA-FFA), used at a non-cytotoxic concentration, is able to counteract the effect of inflammation both on NOTCH1 cascade and cell invasion.

Recently there has been a growing interest in the role of the inflammatory microenvironment in tumorigenesis, and the use of a Conditioned Medium for stimulating epithelial cells has already been employed^41^. This approach aims at evaluating the joint effect of various inflammatory mediators, thus representing a model mimicking *in vivo* scenarios. Notably, it has been previously demonstrated that the cytokines found up-regulated in our CM play a crucial role both in CAC and sporadic CRC^7^. Interestingly, when we applied each single cytokine (IL-6, CXCL8 and TNFα) to CRC cells, we did not observe the same level of NOTCH1 induction obtained through the incubation with CM, although these cytokines were chosen because of their major abundance in CM. We observed a similar result even after combining IL-6, CXCL8 and TNFα, indicating that the complexity of the CM stimulus cannot be reproduced only by three cytokines. Indeed, in this study, we revealed that CM was able to up-regulate the NOTCH1 pathway in HT29 and HCT116 cells. Similarly, Lin and colleagues showed a marked increase of NOTCH1 by incubating CRC cells in a Conditioned Medium collected from cultured mesenchymal stem cells. However, although they observed an increase of full length NOTCH1 expression upon IL-6 treatment, our data show that the level of the active domain of NOTCH1 protein is not affected by this cytokine^23^. The induction of NOTCH1 pathway in our model is confirmed by the concordant modulation of its downstream targets *NRARP* and *ATOH1*, while no changes were observed for HES1, in agreement with the finding that it can be also controlled by NOTCH-independent signalling pathways^42^.

Several reports have indicated a pro-tumorigenic role of NOTCH1 in relation to inflammation in models of sporadic CRC. It has been reported that the crosstalk between NOTCH and IKKα signalling, which plays an essential role in inflammation, exacerbates the cell proliferation and inhibits the apoptosis in HT29, HCT116, CaCo2 and SW480 cell lines^43^. Moreover, the blockage of NOTCH1 cascade has a protective effect toward CRC development in the Apc(Min/+) mouse model of FAP^20^. Otherwise, our research group has recently found a NOTCH1 signalling down-regulation in the azoxymethane-dextran sodium sulfate (AOM-DSS) model of CAC, probably as effect of the epithelial damage due to DSS administration^44^. Interestingly, these opposing effects of inflammation on NOTCH1 signalling might underlie different mechanisms through which inflammation drives cancer development during sporadic CRC and CAC.

Then, we found that CM is able to induce EMT in CRC cells, by regulating both the protein and RNA levels of ZEB1 and MMP9. Recently, Chanrion and colleagues found that the activation of NOTCH and the concomitant p53 deletion triggered EMT in the mouse gut^45^. Since HCT116 and HT29 have respectively wild type and mutant p53, our data indicate that the inflammation-driven EMT mechanism is p53 independent.

Although it is well established that NOTCH signalling can be involved in the induction of EMT^46^, less is known about the possible role of EMT as activator of NOTCH1. Importantly, we show that, in our model, NOTCH1 pathway activation is a consequence of the CM-driven MMP9 overexpression. Garg and colleagues have recently shown that MMP9 up-regulation results in augmented levels of NICD in the AOM-DSS mouse model of CAC, but they attribute a protective role to this mechanism, in accordance with our previous results of NOTCH1 signalling in AOM-DSS treated mice^21^. In contrast, as already discussed, the present study could mimic the effect of the inflammatory *milieu* on sporadic CRC, probably explaining these opposite results.

Eicosapentaenoic Acid (EPA), belonging to the family of long chain Omega-3 polyunsaturated fatty acids (ω-3 PUFAs), is known for its possible role in the prevention and treatment of both inflammatory diseases and CRC^47^, through the modulation of several molecular pathways^35^. In particular, we previously demonstrated that the dietary supplementation of EPA, used as a free fatty acid in an extra pure formula with a higher level of bioavailability (EPA-FFA), significantly protected from cachexia and dramatically suppressed polyp number, size and burden in Apc(Min/+) mice, by inhibiting COX-2 expression, and reducing β-catenin nuclear translocation, cell proliferation, together with an increase in apoptosis^37^. However, many other unknown pathways could be involved in the action of EPA. In the present study, we performed a prolonged pre-treatment with EPA-FFA both for 72 h or 14 days on CRC cells. We found that the incorporation of EPA into the cellular membrane was similar in these conditions, indicating that the maximum incorporation was reached very quickly due to the FFA formulation. Noteworthy, we show for the first time that EPA-FFA is able to counteract the up-regulation of NICD and JAG1 in HT29 and HCT116 cells and tends to inhibit the MMP9 activity in HCT116. Moreover, EPA-FFA opposes the CM-induced cellular invasiveness both in HT29 and HCT116, whereas HCT116 cells are known to exhibit higher invasion capability than HT29 cells^48^. Similarly, Cheng-Chung Li and his group found that a treatment with EPA and DHA decreased MMP9 level in prostate cancer cells^49^. It is important to note that although the above-mentioned differences between our previous AOM-DSS *in vivo* work and the present *in vitro* study, EPA-FFA showed the same capability to modulate NOTCH1 signalling in both models^44^.

Taken together, our data demonstrate for the first time that inflammation is a strong activator of the NOTCH1 cascade through overexpression of MMP9 and that EPA-FFA could exert a preventive action against this mechanism in CRC.

## Material and Methods

### Cell lines and treatments

The human colorectal cancer cell lines HT29, HCT116, LS174T and the human monocyte cell line THP-1 were obtained from ATCC (Manassas, VA, USA). In our laboratory, all cell lines are tested and authenticated every year using known genetic and epigenetic markers. HT29 and THP-1 cell lines were cultured in RPMI (with the addition of 50 μM of β-mercaptoethanol for THP-1), while HCT116 and LS174T cell lines were grown in Iscove’s Modified Dulbecco Media (IMDM). The culture media were supplemented with 10% FBS (20% for THP-1), 100 U/ml penicillin, 100 μg/ml streptomycin and 2 mM glutamine (Euroclone, Milan, Italy). The cells were maintained at 37 °C and 5% CO_2_.

HT29, HCT116 and LS174T cell lines were exposed to CM for 12 h. HT29 cells were also incubated with 100 ng/ml of interleukin-6 (IL-6, Immunological Sciences, Roma, Italy), 1 μg/ml of chemokine-8 (CXCL8, Sigma Aldrich) or 100 ng/ml of Tumor Necrosis Factor-alpha (TNF-α, PeproTech Inc., Rocky Hill, United States), or their combination for 72 h.

Eicosapentaenoic acid as free fatty acid (EPA-FFA, ALFA, SLA Pharma AG, Switzerland) was used for cell culture treatments both at 72 h and 14 days. The cells were pre-treated with 50 μM of EPA-FFA for 72 h prior to CM incubation.

### Conditioned Medium (CM) production and characterization

THP-1 were differentiated into macrophages using 5 ng/ml of phorbol 12-myristate 13-acetate (PMA, Sigma Aldrich, Milan, Italy) for 72 h. The differentiation of THP-1 was assessed by determining the CD14 and CD11b positive cell population. Briefly, 0.5 × 10^6^ PMA-treated THP-1 cells were washed with PBS and incubated with PerCP-conjugated anti-CD14 (Miltenyi Biotec, Bologna, Italy) and PE-conjugated anti-CD11b (Exalpha Biologicals, MA, USA) in PBS supplemented with 1% BSA on ice for 30 min. Then, the cells were analysed with a FACSaria flow cytometer (BD Biosciences, Milano, Italy). After differentiation, the medium containing PMA was removed, THP-1 were washed with PBS and incubated in RPMI supplemented with 50 ng/ml of E.coli lipopolysaccharide (LPS, strain 055:B5, Sigma Aldrich) for 1 h. Then, the medium containing LPS was replaced with fresh medium in order to obtain an LPS-free, cytokine-enriched conditioned medium (CM)^50^,^51^. After 24 h, the CM was collected, filtered and stored at −80 °C until use.

The CM composition was characterized by measuring the concentrations of the major cytokines: interleukin (IL)-1β, IL-2, IL-4, IL-6, CXCL-8, IL-10, IL-12, IL-17, interferon-γ (IFNγ), monocyte chemoattractant protein-1 (MCP-1), macrophage inflammatory protein 1-β (MIP1-β), tumor necrosis factor-α (TNFα), granulocyte-colony stimulating factor (G-CSF), granulocyte-macrophage colony-stimulating factor (GM-CSF) with a multiplex bead-based sandwich immunoassay (Bio-Plex, Bio-Rad Laboratories, Milan, Italy), following the manufacturer’s instructions. Two replicates were analyzed for each sample.

### Western Blot

Eighty μg of proteins for each sample were separated on a 4–12% SDS-polyacrylamide gel (Life Technologies, Monza, Italy) and transferred onto nitrocellulose membrane. After being blocked with nonfat dry milk, the membranes were incubated overnight at +4 °C with the following primary antibodies: mouse monoclonal anti-NOTCH1 1:1000 (clone mN1A, catalog number 629101, Biolegend, SA, USA), rabbit polyclonal anti-cleaved NOTCH1 1:500 (Val1744, catalog number 2421, Cell Signaling Technologies, Leiden, Netherlands), rabbit polyclonal anti-JAG1 1:200 (clone H-114, catalog number sc-8303, Santa Cruz Biotechnology, Heidelberg, Germany), rabbit monoclonal anti-ZEB1 (anti-TCF8/ZEB1, 1:1000, clone: D80D3, Cell Signaling), rabbit polyclonal anti-MMP9 (1:1000, catalog number: 3852, Cell Signaling), and rabbit monoclonal anti-CDH1 (1:1000, clone: 24E10, catalog number: 3852, Cell Signaling). Following incubation with the appropriate secondary antibody, the signal was detected with Chemiluminescent Sensitive Plus HRP (BioFx Laboratories, MD, United States), and images were acquired with the ChemidocTM XRS+ (Bio-Rad Laboratories). Mouse monoclonal anti-βACTIN (1:2000, clone AC-15, catalog number A1978, Sigma Aldrich) was used as housekeeping protein. Each experiment was repeated at least twice.

### RNA extraction and quantitative RT-PCR (qRT-PCR)

RNA was isolated using Trizol reagent (Life Technologies, Monza, Italy) and reverse transcribed using GoScript^TM^ Reverse Transcriptase Kit (Promega, Milan, Italy). qRT-PCR was performed using TaqMan Gene Expression Assays for *HES1* (Hs00172878_m1), *ATOH1* (Hs0094192_s1), *GAPDH* (Hs99999905_m1) or Sybr select Master mix for *JAG1*, *NRARP*, *ZEB1*, *MMP9* and *CDH1* (Life Technologies). Primer sequences for Sybr green assays are reported in Supplementary Table S1. qRT-PCR was performed on iCycler (Bio-Rad Laboratories) and the data were analysed by means of the 2^−∆∆Ct^ method. At least two replicates were analysed for each sample.

### Membrane fatty acid analysis

The amount of membrane fatty acids was determined by GC-MS as previously described^36^. Fatty acid levels were expressed as relative percentages of total fatty acids. Heptadecanoic acid (17:0) was used as internal standard.

### Cell viability assay

HT29 and HCT116 cells were seeded into 96-well plates (5 and 3 × 10^3^ cells, respectively) and treated with different concentrations of EPA-FFA (0–200 μM). After 72 h of treatment, cell viability was measured using the 3-(4, 5-dimethylthiazol-2-yl)-2, 5-diphenyltetrazolium bromide assay (MTT, Sigma Aldrich) following the manufacturer’s instructions. Each experiment was performed in quintuplicate.

### NOTCH1 stable silencing

pSuper.puro expression vectors with short hairpin oligonucleotides targeting NOTCH1 exons were kindly provided by Dr. Catia Giovannini. The retroviruses obtained by the transient transfection of pSuper.puro vectors into Phoenix A packaging cells were used to transduce the HT29 cells. The HT29 NOTCH1^−/−^ cells were selected in RPMI supplemented with 2 μg/ml of puromycin.

### MMP9 transient silencing

HT29 cells were transfected with 50 nM of small interfering RNA (siRNA) against MMP9 (ON-TARGETplus SMARTpool siRNA sequences, Dharmacon, Thermo Fisher Scientific, Cultek SL, Madrid, Spain) for 48 h using Dharmafect transfection reagent-1 (Thermo Fisher Scientific, T-2001-01) according to the manufacturer’s instructions. A nontargeting siRNA (D-001810-10-05, Dharmacon) was used as a negative control (scramble).

### Invasion assay

Cell invasion was assessed by Boyden blind-well chambers (New Technologies Group, Monza e Brianza, Italy) containing polycarbonate filters with 8-μm pore, coated with Matrigel (Sigma Aldrich). After CM and/or EPA-FFA treatment, 7 × 10^4^ HT29 and HCT116 cells were resuspended in serum-free medium and added to the upper chamber. A medium supplemented with 10% FBS was used as chemoattractant in the lower chamber. Cells were incubated at 37 °C for 12 h and non invading cells were removed with cotton swabs. The cells adhering to the under-side of the filter were fixed, stained with Giemsa (Sigma Aldrich) and counted under microscope (10 fields per chamber).

### Gelatin Zymography

MMP9 activity was determined by gelatin zymography as previously described^52^ with minor modifications. Briefly, cells were treated with CM for 12 h, then CM was removed and cells were incubated o.n. in serum-free medium. The media was collected and centrifuged (400 g, 5 min at 4 °C) to remove cells and debris. Proteins were precipitated with 1:4 (vol/vol) ice-cold methanol o.n. at −20 °C, solubilized with sample buffer and 20 μg were loaded in 10% polyacrylamide gels co-polymerized with 1% gelatin. After electrophoresis, the gels were washed twice in 2.5% Triton X-100 and incubated o.n. in an activation buffer (50 mM Tris–HCl supplemented with 5 mM CaCl2). The gels were stained with Comassie brilliant blue R-250 and de-stained with 50% methanol and 10% acetic acid in distilled water. MMP9 proteolytic activity was quantified using ChemiDoc^TM^ XRS+(Biorad, CA, USA).

### Statistical analysis

Data were analyzed with Graphpad 5.0 Software (GraphPad Software Inc., CA, USA). Whenever necessary, the values were transformed using the function Y = log(Y) to stabilize the variances. The means of two unmatched groups were compared using the unpaired T test, while the one-way ANOVA followed by Tukey’s or Dunnet’s post hoc tests were used to compare the means of three or more groups. The data are shown as Mean ± SEM. P values less than 0.05 were regarded as statistically significant.

## Additional Information

**How to cite this article**: Fazio, C. *et al.* Inflammation increases NOTCH1 activity via MMP9 and is counteracted by Eicosapentaenoic Acid-free fatty acid in colon cancer cells. *Sci. Rep.* **6**, 20670; doi: 10.1038/srep20670 (2016).

## Supplementary Material

Supplementary Information
