# Supplementary material for: Inflammation increases NOTCH1 activity via MMP9 and is counteracted by Eicosapentaenoic Acid-free fatty acid in colon cancer cells
Source: Sci Rep. 2016 Feb 11;6:20670. doi: 10.1038/srep20670 (PMC4749954; doi:10.1038/srep20670)
Supplement: Supplementary Information [file srep20670-s1.pdf]

Supplementary Tables and Figures

Inflammation increases NOTCH1 activity via MMP9 and is counteracted by Eicosapentaenoic Acid-free fatty acid in colon cancer cells

Chiara Fazio, Giulia Piazzi, Paola Vitaglione, Vincenzo Fogliano, Alessandra Munarini, Anna Prossomariti, Maddalena Milazzo, Leonarda D'Angelo, Manuela Napolitano, Pasquale Chieco, Andrea Belluzzi, Franco Bazzoli and Luigi Ricciardiello

Table S1: Primer sequences

Gene	Accession n.	Forward Primers (5'-3')	Reverse Primers (5'-3')
Jagged1	NM_000214	AAGGCTTCACGGGAACATAC	AGCCGTCACTACAGATGCAC
NRARP	NM_001004354	GGGCTGCATAGAAAATTGGA	CCCTTTTTAGCCTCCCAGAG
ZEB1	XM_006717499.1	TACAGAACCCAACTTGATCGTCACA	GATTACACCCAGACTGCGTCACA
MMP9	NM_004994.2	TTGACAGCGACAAGAAGTGG	GCCATTCACGTCGTCCTTAT
E-Cadherin	NM_004360.3	GCCGCTGGCGTCTGTAGGAA	TGACCACCGCTCACCTCCGA

S1

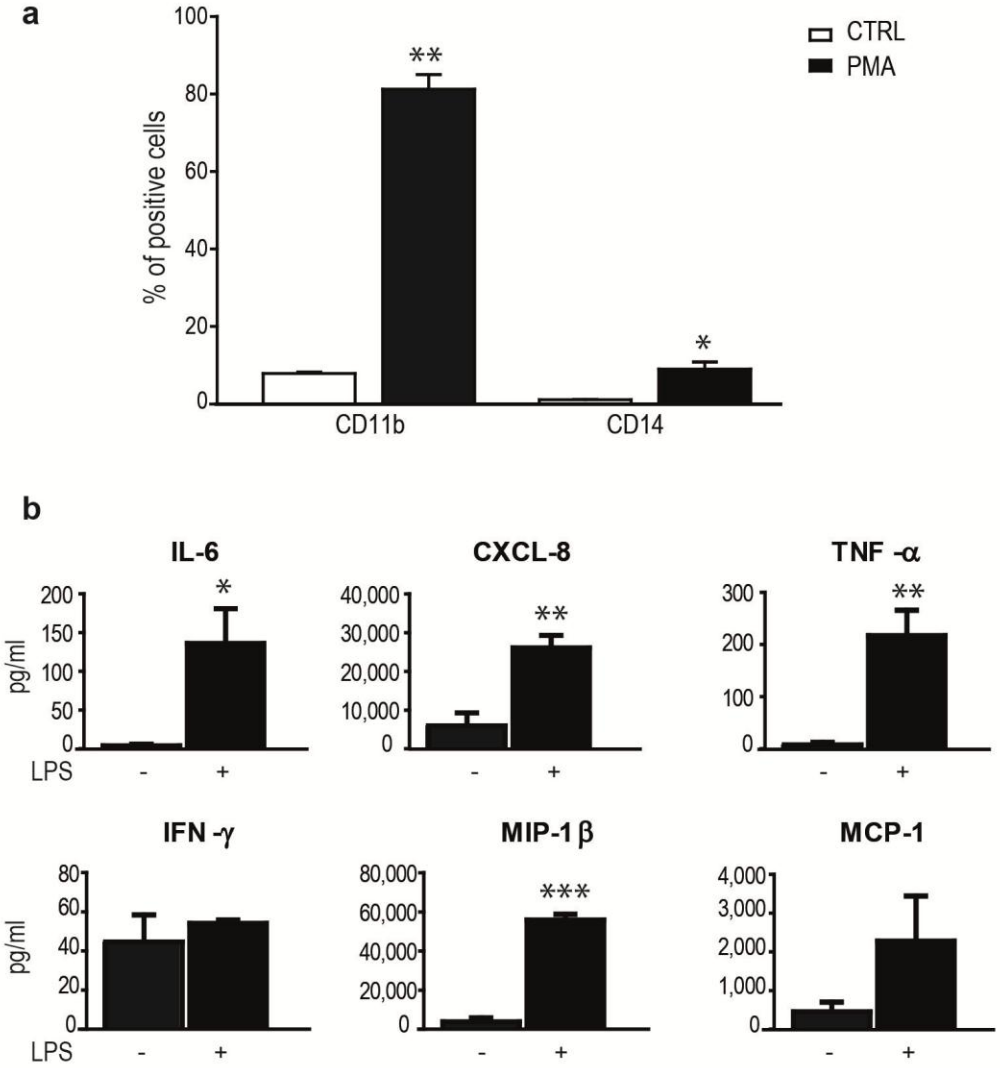

Supplementary Fig. S1. (A) FACS analysis for the expression of CD11b and CD14 in THP1 cells untreated (Ctrl) or treated with 5ng/ml PMA. **(B)** Pro-inflammatory mediators in the conditioned medium (CM) from PMA-differentiated THP1 before and after activation with 50 ng/ml of LPS for 1h. Two replicates were analysed for each sample. Number of replicates (n)=2, number of independent experiments (N)=2. *= $p<0.05$, **=$p<0.01$, ***=$p<0.001$, unpaired t-test.

S2

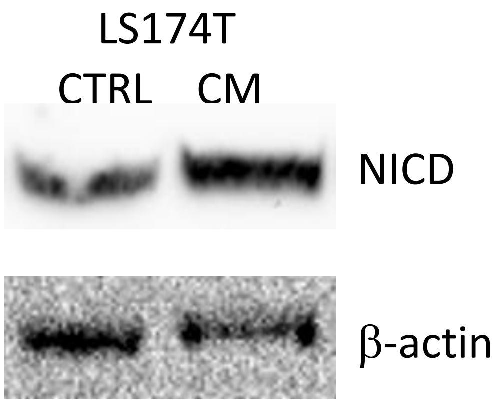

Supplementary Fig. S2. Western Blot for NICD in LS174T cells treated with CM. β-actin was used as housekeeping protein.

S3

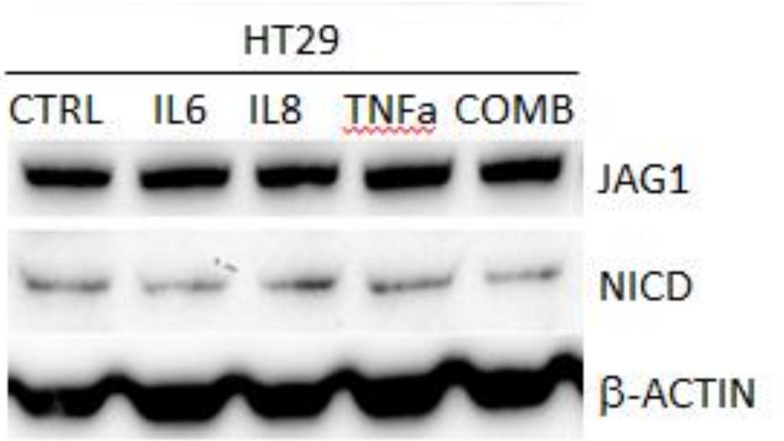

Supplementary Fig. S3. Western Blot analyses for Jagged1 and NICD in HT29 treated with IL-6 (100 ng/ml) CXCL-8 (1μg/ml) or TNF-α (100 ng/ml) or the combination of all (COMB). βactin was used as housekeeping protein

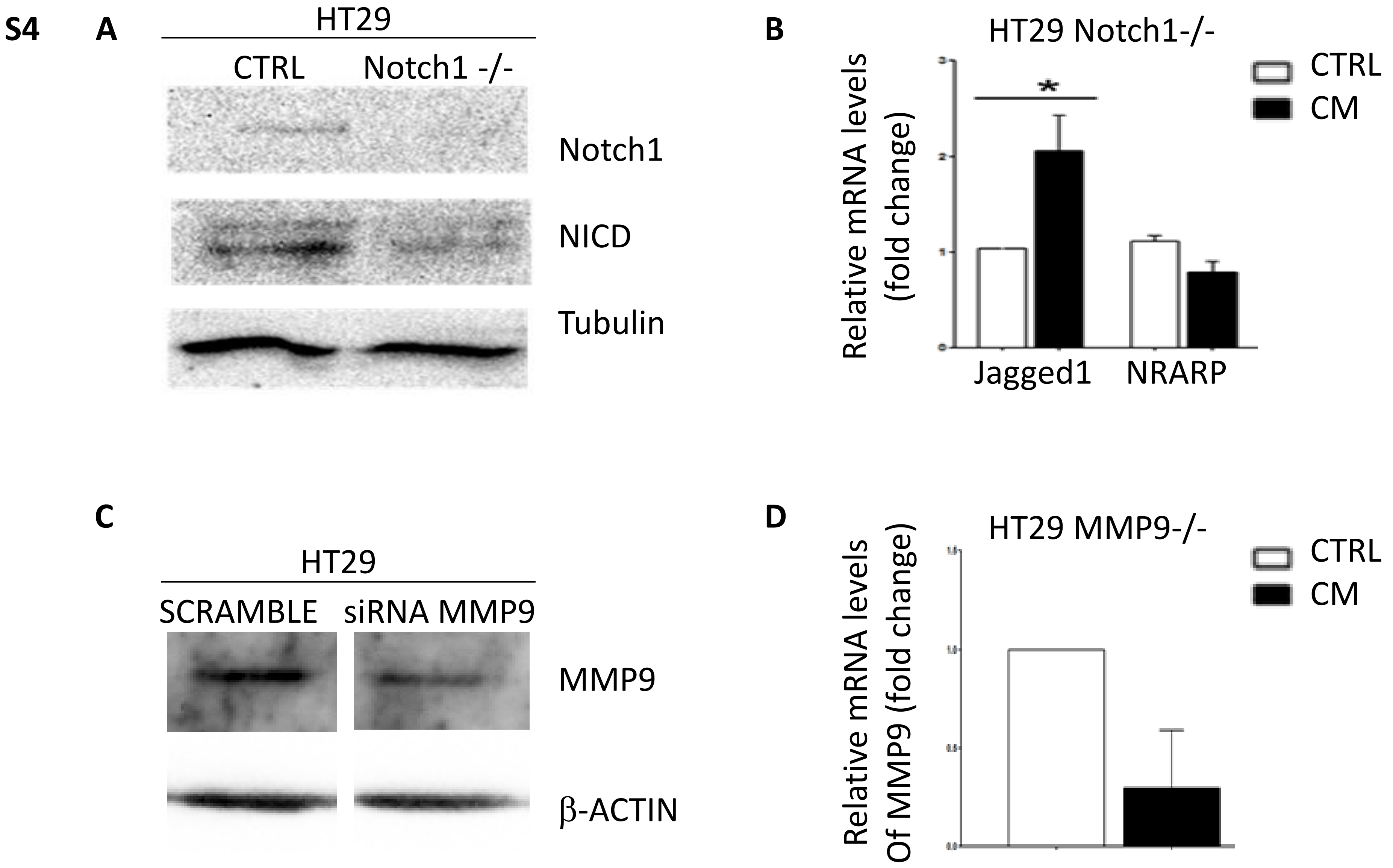

Supplementary Fig. S4.(A) Western Blot analyses for Notch1 Full Length, NICD in HT29 CTRL and HT29 Notch1-/- **(B)** qRT-PCR for Jagged1 and NRARP in HT29 Notch1-/- CTRL and HT29 Notch1-/-+CM **(C)** Western Blot analyses for MMP9 in HT29 SCRAMBLE and HT29 MMP9-/- **(D)** qRT-PCR for MMP9 in HT29 MMP9-/- CTRL and HT29 MMP9-/-+CM

S5

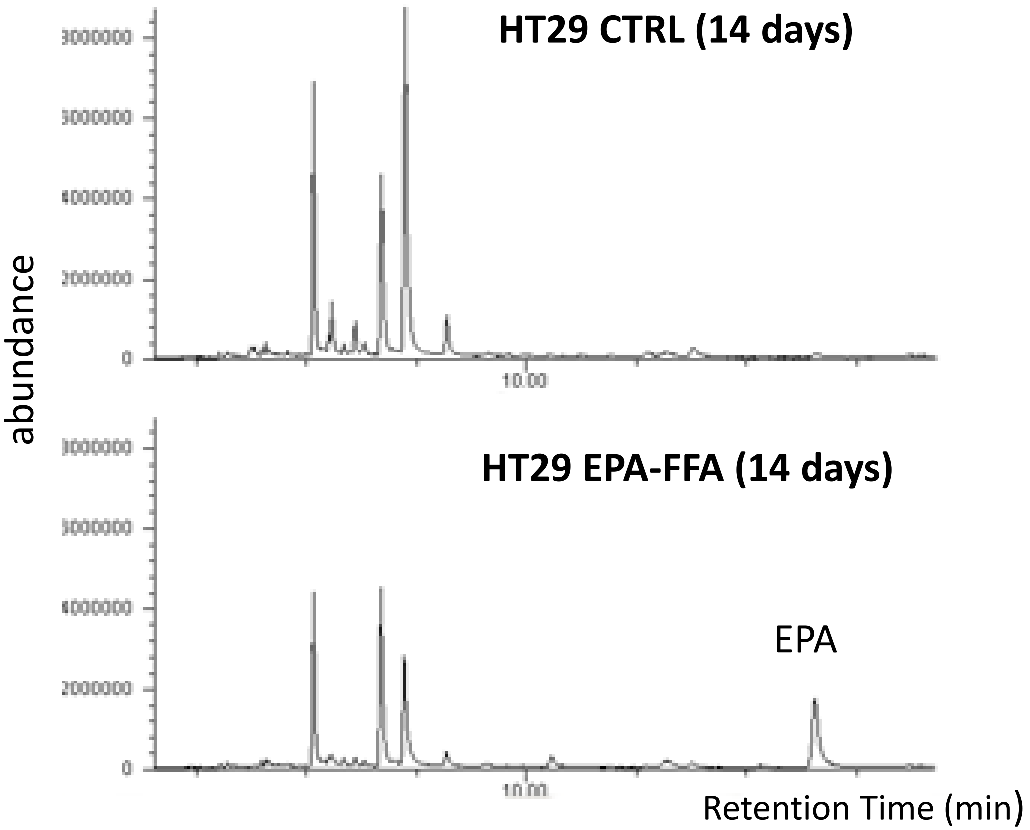

Supplementary Fig. S5. Spectrum of peaks from Gas chromatography-mass spectrometry (GC/MS) analysis of HT29 CTRL (upper panel) and 14 days-EPA treated HT29 cells (lower panel)
